# How artificial intelligence is reshaping citation impact in oral and maxillofacial radiology journals: an 8-year analysis with editorial and clinical implications (JCR 2017–2024)

**DOI:** 10.3389/frma.2026.1815503

**Published:** 2026-05-08

**Authors:** Andy Wai Kan Yeung, Deepal Haresh Ajmera, Andrew Nalley, Ray Tanaka, Kuo Feng Hung, Michael M. Bornstein

**Affiliations:** 1 Oral and Maxillofacial Radiology, Applied Oral Sciences and Community Dental Care, Faculty of Dentistry, The University of Hong Kong, Hong Kong, China; 2 Department of Oral Health &; Medicine, University Center for Dental Medicine Basel UZB, University of Basel, Basel, Switzerland

**Keywords:** artificial intelligence, cone-beam CT, dental imaging, editorial policy, journal impact factor, scientometrics

## Abstract

**Objectives:**

To quantify how artificial intelligence (AI) publications contribute to journal impact factor (JIF) in oral and maxillofacial radiology (OMFR) journals and to discuss implications for imaging research, peer review, and clinical translation.

**Methods:**

On 25 June 2025, Journal Citation Reports (JCR) data (2017–2024) were retrieved for Dentomaxillofacial Radiology, Oral Radiology, Imaging Science in Dentistry, and the Radiology section of Oral Surgery, Oral Medicine, Oral Pathology and Oral Radiology (OOOO). For each JCR year, citable items and JIF-accountable citations were exported and manually coded as AI article, AI review, non-AI article, or non-AI review by two observers (κ = 0.956). A descriptive indicator, named as notional JIF, was computed to illustrate the contribution of AI items. Citation rates were compared descriptively across document types.

**Results:**

In JCR 2024, AI papers represented 10.9%−25.2% of citable items among OMFR journals yet contributed 31.3%−53.7% of JIF-accountable citations; radiology AI papers contributed materially to OOOO's JIF despite comprising only 1.5%−2.4% of citable items. AI articles and reviews received 4.3–4.4 × more JIF-accountable citations per item than non-AI counterparts. Notional JIFs exceeded actual JIFs in 2020–2022, reflecting small-denominator effects and topic-specific citation acceleration that diminished as more AI papers were published in subsequent years.

**Conclusions:**

AI-related publications are associated with higher per-item citation rates in OMFR journals, consistent with recognized patterns of topic-focused citation concentration in fast-moving research areas. These descriptive findings highlight the importance of methodological transparency, robust validation practices, and balanced editorial policies as AI research continues to expand.

## 1 Introduction

Artificial intelligence (AI) has rapidly become a cornerstone of innovation in healthcare, with dental medicine being no exception. The integration and continuing development of AI technologies has enabled transformative changes in diagnostic imaging, clinical decision making support, and patient management (Joda et al., 2021). In oral and maxillofacial radiology (OMFR), AI applications have shown particular promise in automating image interpretation, detecting pathologies, and enhancing diagnostic accuracy, thereby improving both efficiency and outcomes in clinical practice (Hung et al., 2020a,b, 2022a, 2023).

This technological evolution has been accompanied by a surge in scholarly interest, as evidenced by the growing number of AI-related publications in dental journals (Lu et al., 2025). The increasing prevalence of such research reflects not only the enthusiasm of the scientific community for this field but also the broader shift toward data-driven and algorithm-enhanced healthcare (Hung et al., 2022b). Importantly, this trend raises questions about how AI-related publications are contributing to traditional metrics of academic impact, such as the journal impact factor (JIF), which remains a key indicator of journal prestige and visibility.

Multiple recent studies have explored the rise of AI in dental and medical literature. Thurzo et al. (2022) found that the annual growth of AI publications in dentistry since 2018 has been faster than before, reaching at a rate of 34.9% increase per year. Feher et al. (2024) highlighted the diversity of AI applications in dentistry, including diagnostics and risk stratification, whereas Allani et al. (2024) observed a marked increase in AI publications post-2018, particularly on diagnosis of caries. In broader healthcare contexts, Guo et al. (2020) and Lin et al. (2025) also reported exponential growth in medical AI-related publications and citations. These studies collectively illustrate the growing academic impact of AI research especially in dental medicine, but none have specifically quantified its contribution to journal metrics in OMFR. Beyond dentistry, several non-dental bibliometric analyses have documented similar increase in the interest for AI research in medicine and healthcare, such as a 59% increase in AI publications within healthcare and public health literature over 2015–2025 (Ettadili and Darvishmohammadi, 2025), and an annual publication growth of 28.4% of AI research in medicine especially across precision medicine, digital health, and COVID-19-related research during 2019–2023 (Lin et al., 2025). These reports suggest that AI-driven publication or citation concentration is a broad cross-disciplinary pattern rather than a dentistry-specific phenomenon.

As adoption grows, editors, reviewers, clinicians, and methodologists need robust, field-specific evidence about how AI research circulates and concentrates influence within specialist journals. Beyond performance metrics of individual algorithms, publication and citation behaviors provide a complementary lens on the maturity, reach, and perceived utility of AI methods in OMFR. This study addresses that gap by analyzing the publication and citation patterns of AI-related papers in three dedicated OMFR journals, namely Dentomaxillofacial Radiology (DMFR), Oral Radiology, and Imaging Science in Dentistry; and one multidisciplinary journal considered important in OMFR research field, namely Oral Surgery, Oral Medicine, Oral Pathology and Oral Radiology (OOOO). The present study therefore aims to describe the association between AI-related publications and JIF-accountable citations in OMFR journals. Our objective is not to infer causality, but rather to document citation patterns, highlight topic-specific citation concentration phenomena, and provide observations useful for editors, reviewers, and researchers.

## 2 Materials and methods

On 25 June, 2025, the Journal Citation Reports (JCR) electronic database was accessed. We analyzed all indexed OMFR-dedicated journals listed in JCR during the study window (DMFR, Oral Radiology, Imaging Science in Dentistry). OOOO's Radiology section was included because it represents the only mixed-discipline journal with a sizeable OMFR research component. Thus, the dataset represents the complete population of OMFR journals in JCR rather than a selective sample.

**Figure 1:**
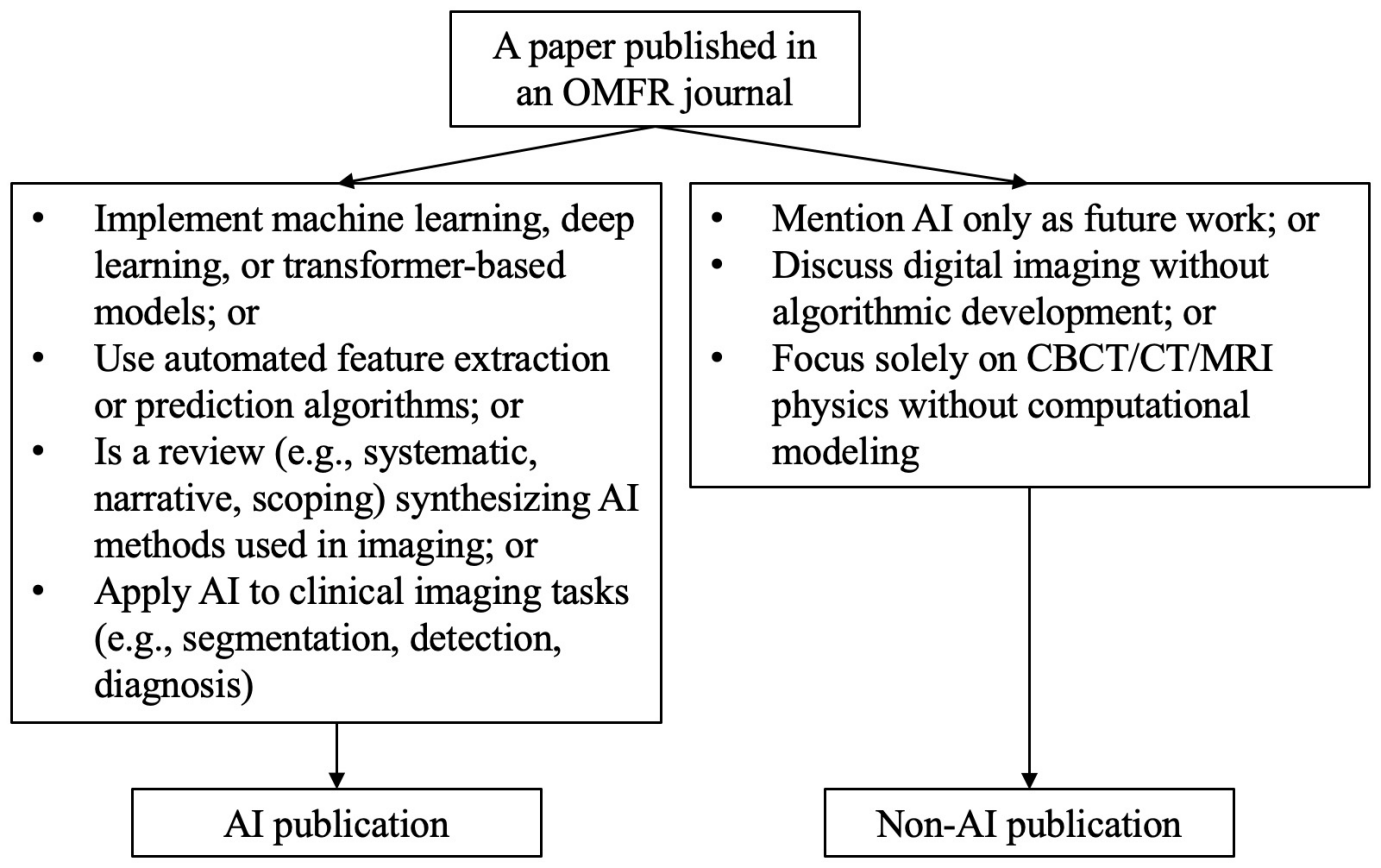
Flow diagram that illustrates the classification decisions of the analyzed publications.

The JIF data of the four journals were exported per JCR year, including the list of citable items and their citation count accountable to JIF calculations. The list of citable items and JIF-accountable citations are available for JCR year 2017 onwards. From default definition, citable items are either articles or reviews. Readers should note that the data readily available from JCR is for the calculation of the widely used 2-year JIF, but not for 5-year JIF. As past literature has indicated a rapid growth of the dental research publications in AI since 2018–2020 (Thurzo et al., 2022; Allani et al., 2024; Lu et al., 2025), the current study covered the JCR period from 2017 to 2024, meaning that citable items published since 2015 were included. Imaging Science in Dentistry received its first JIF in 2022, and therefore its data from 2022 to 2024 only was collected. Each citable item was manually coded by two observers (DHA and AWKY) as a paper dealing with AI topics (AI paper) or non-AI paper according to its title, abstract and keywords. For OOOO, only papers published under the Radiology section were coded and therefore results should be considered as exploratory. Both observers assessed the entire dataset. In particular, the observers looked for AI-related terms (e.g., AI, machine learning, deep learning, convolutional neural network, transformer, generative adversarial networks, natural language processing, large language models, chatbots, vision-language models, radiomics, supervised learning, semi-supervised learning, unsupervised learning, and so on) and determined if a paper involved AI research or not. For example, a paper would be classified as non-AI if it only mentioned AI should be used in future studies. A flow diagram is shown in Figure 1 to illustrate the classification decisions. Disagreements were resolved by mutual consensus of the two observers after discussion. The overall inter-observer agreement was high as assessed by Cohen's kappa coefficient (κ = 0.956). Besides AI vs. non-AI, an AI publication could be further categorized into image-based AI, text-based AI, and a hybrid of them. However, since most of the OMFR AI papers were image-based, such categorization and subsequent analyses were not performed.

To illustrate the impact on the annual JIF of the OMFR journals, a “notional” JIF was computed by dividing the number of JIF-accountable citations received by AI papers with the number of AI papers (Table 1). It is not intended as an evaluative metric, but to illustrate how AI topic-specific citation clustering affects journal-level metrics. Raw numerator and denominator as well as confidence intervals for each notional JIF are provided in Supplementary Table S1. This data would be visualized alongside the journals' actual JIF. Meanwhile, the ratio of AI papers and the ratio of their JIF contribution (i.e. the ratio of JIF-accountable citations) per JCR year would be computed and visualized. Finally, the annual data of the number of JIF-accountable citations per citable item for four paper types (AI original article, AI review, non-AI article, and non-AI review) were computed. For OOOO, only papers published under the Radiology section were considered, as described above.

**Table 1:** Definitions of journal impact factor (JIF) and notional JIF.

Term used in this study	Definition
Journal impact factor (JIF) of JCR year 2024	$\frac{Citations\;in\;2024\;to\;items\;published\;in\;2022\;+\;2023}{Number\;of\;citable\;items\;in\;2022\;+\;2023}$
Notional JIF of JCR year 2024	$\frac{Citations\;in\;2024\;to\;AI\;radiology\;papers\;published\;in\;2022\;+\;2023}{Number\;of\;AI\;radiology\;papers\;in\;2022\;+\;2023}$

Two-way ANOVAs were conducted to test if AI involvement (AI vs. non-AI) and document type (article vs. review) were significant factors in associating with the JIF-accountable citations per citable item for each JCR year.

Statistical analysis was conducted with SPSS Version 28.0 (IBM, NY, USA). Results were considered statistically significant if *P* < 0.05. Since this study did not involve human or animal data, ethical approval was not applicable.

## 3 Results

**Figure 2:**
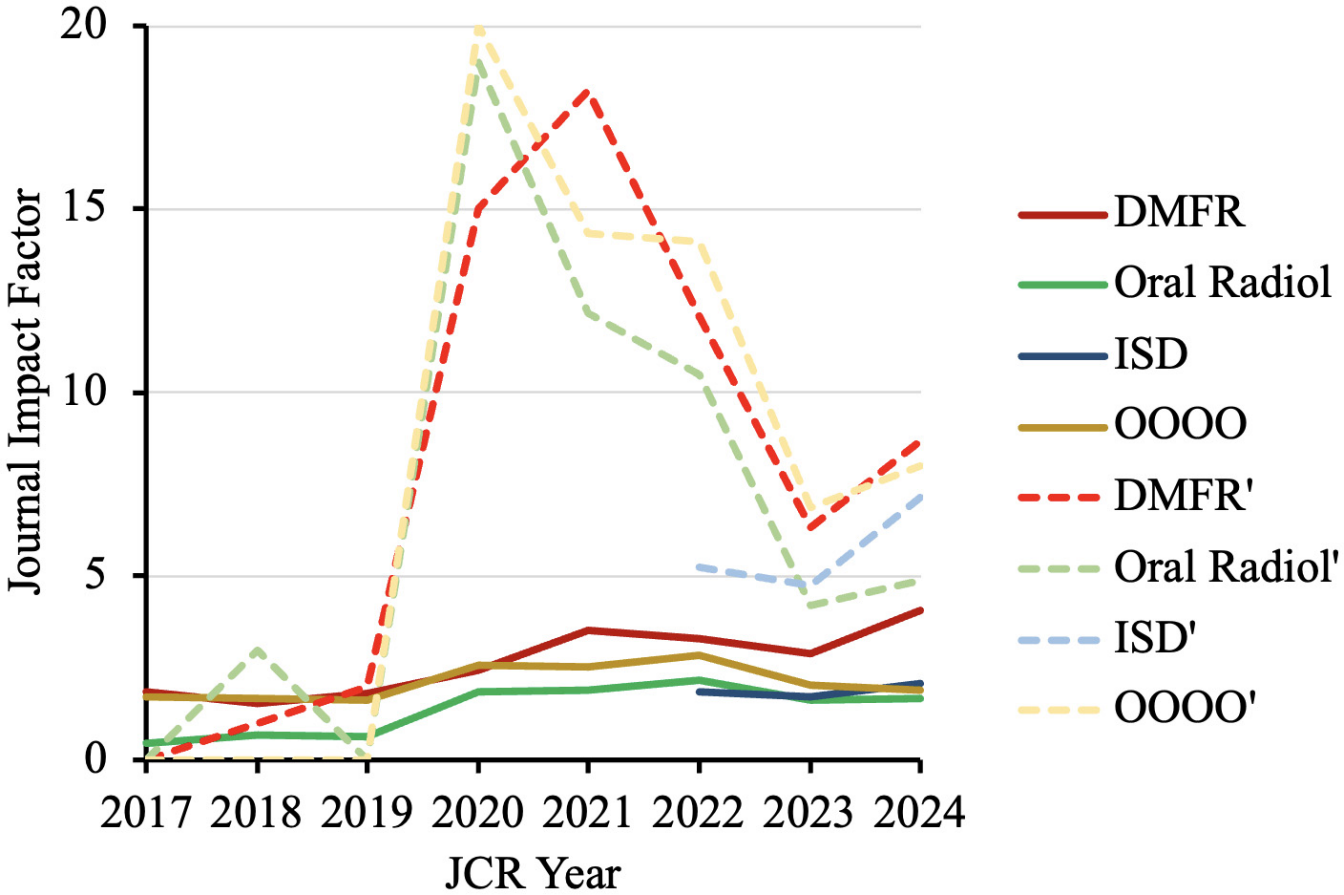
Journal impact factors (JIFs; solid lines) of oral and maxillofacial radiology journals and their notional JIFs (denoted by an apostrophe; dotted lines) if only AI radiology papers were considered. DMFR, Dentomaxillofacial Radiology; Oral Radiol, Oral Radiology; ISD, Imaging Science in Dentistry; OOOO, Oral Surgery, Oral Medicine, Oral Pathology and Oral Radiology. Please see Supplementary Table S1 for the raw numbers used to compute the notional JIFs and their confidence intervals.

Across 2017–2024, all journals increased their JIFs (Figure 2). From 2020 onward, AI-only notional JIFs markedly exceeded actual values, indicating a concentrated citation rise by AI content. For instance, the notional JIF of Dentomaxillofacial Radiology, Oral Radiology, and OOOO in 2020 could reach 15.0, 19.0, and 20.0, respectively. In other words, the notional JIF were 6.20–10.26 times of the actual values. Readers should note that the notional JIF may be inflated due to small number of AI papers. Please refer to Supplementary Table S1 for the numerator and denominator as well as 95% confidence intervals for each notional JIF.

**Figure 3:**
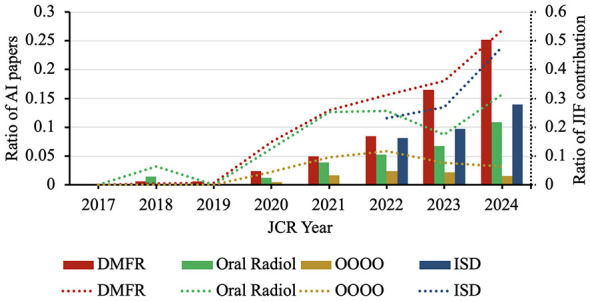
Ratio of AI radiology papers (bars) and their contributions to journal impact factor (JIF; dotted lines) of the four oral and maxillofacial radiology journals. DMFR, Dentomaxillofacial Radiology; Oral Radiol, Oral Radiology; ISD, Imaging Science in Dentistry; OOOO, Oral Surgery, Oral Medicine, Oral Pathology and Oral Radiology. For OOOO, the ratio of AI papers referred to the ratio of AI radiology papers to the total number of citable items.

There were very few or even no AI papers published in OMFR journals that could contribute to the calculation of JIF during JCR years 2017–2019 (Figure 3). Since 2020, the ratio of AI papers began to increase year by year, and their ratio of JIF contribution (i.e., ratio of JIF-accountable citations) showed an overall upward trend.

In JCR 2024, AI papers accounted for 10.9%−25.2% of citable items in the three pure OMFR journals yet contributed 31.3%−53.7% of JIF-accountable citations. For OOOO, radiology AI papers constituted 1.5%−2.4% of citable items but supplied 6.5%−11.8% of JIF-accountable citations during the period of 2021–2024.

Starting from JCR 2020, AI papers have had higher JIF-accountable citations per citable item than non-AI counterparts (Table 2). In JCR 2024, AI articles and AI reviews received approximately 4.4 × and 4.3 × more JIF-accountable citations than non-AI articles and reviews, respectively. Table 3 lists the top 10 citable items with the highest number of JIF-accountable citations per journal over the survey period; most of them are AI papers.

**Table 2:** Breakdown of journal impact factor (JIF)-accountable citations per citable item by Journal Citation Reports (JCR) year, according to involvement of AI and document type.

Citable item type	JIF-accountable citations per citable item by JCR year, mean (SD)							
	2017	2018	2019	2020	2021	2022	2023	2024
AI article	NA	2.00 (1.41)	2.00 (NA)	17.00 (6.03)	14.47 (10.07)	10.14 (8.91)	5.21 (3.11)	7.03 (5.07)
AI review	NA	NA	NA	NA	30.00 (NA)	14.67 (17.55)	9.13 (6.15)	10.25 (9.51)
Non-AI article	1.03 (1.54)	1.21 (1.45)	1.28 (1.74)	1.89 (2.23)	1.77 (1.99)	1.71 (2.01)	1.49 (1.86)	1.58 (1.79)
Non-AI review	6.13 (8.36)	1.96 (2.44)	1.52 (1.72)	2.26 (2.53)	3.45 (3.43)	2.73 (3.34)	2.42 (2.74)	2.37 (2.78)
*P* value (AI involvement)	NA	0.474	0.682	4.4248E-45	3.9689E-30	1.1217E-31	5.8478E-28	7.2402E-33
Effect size (AI involvement)	NA	0.002	0.001	0.456	0.280	0.225	0.200	0.263
*P* value (document type)	1.139E-15	0.028	0.496	0.394	9.2667E-8	0.000726	1.0172E-7	0.000118
Effect size (document type)	0.203	0.017	0.002	0.002	0.070	0.021	0.051	0.031
Number of items								
Total no. of citable items	285	293	290	330	400	543	544	471
No. of citable items being AI-related	0	2	1	7	20	41	60	73
Ratio of citable items being AI-related	0	0.007	0.003	0.021	0.050	0.076	0.110	0.155
AI article	0	2	1	7	19	35	52	61
AI review	0	0	0	0	1	6	8	12
Non-AI article	262	268	260	288	333	436	418	352
Non-AI review	23	23	29	35	47	66	66	46

*P* values based on two-way ANOVA (AI × document type). Effect sizes in ANOVA are expressed as partial eta squared. NA, not applicable. Readers should be aware that the yearly data comes from citable items published in the preceding 2 years. For example, the data of JCR year 2024 corresponds to the citations received in 2024 by citable items published in 2022 and 2023; it is not the total citations received by papers published in 2024.

**Table 3:** Top 10 citable items with the highest number of journal impact factor (JIF)-accountable citations during Journal Citation Reports (JCR) years 2017–2024 per OMFR journal.

Citable item	Document type	Publication year	JIF-accountable citations
Dentomaxillofacial Radiology			
The use and performance of artificial intelligence applications in dental and maxillofacial radiology: a systematic review (Hung et al., 2020a)	AI review	2020	80
Tooth detection and numbering in panoramic radiographs using convolutional neural networks (Tuzoff et al., 2019)	AI article	2019	56
Current applications and development of artificial intelligence for digital dental radiography (Putra et al., 2022)	AI review	2022	50
A deep-learning artificial intelligence system for assessment of root morphology of the mandibular first molar on panoramic radiography (Hiraiwa et al., 2019)	AI article	2019	49
Automatic diagnosis for cysts and tumors of both jaws on panoramic radiographs using a deep convolution neural network (Kwon et al., 2020)	AI article	2020	38
Deep learning for automated detection and numbering of permanent teeth on panoramic images (Estai et al., 2022)	AI article	2022	36
Cone beam computed tomography in Dentomaxillofacial Radiology: a two-decade overview (Gaêta-Araujo et al., 2020)	Non-AI review	2020	34
CBCT-based bone quality assessment: are Hounsfield units applicable? (Pauwels et al., 2015)	Non-AI review	2015	32
Performance of deep learning object detection technology in the detection and diagnosis of maxillary sinus lesions on panoramic radiographs (Kuwana et al., 2021)	AI article	2021	31
Artificial intelligence in oral and maxillofacial radiology: what is currently possible? (Heo et al., 2021)	AI review	2021	29
Artificial intelligence system for automatic deciduous tooth detection and numbering in panoramic radiographs (Kilic et al., 2021)	AI article	2021	29
Oral radiology			
Evaluation of an artificial intelligence system for detecting vertical root fracture on panoramic radiography (Fukuda et al., 2020b)	AI article	2020	57
Deep-learning classification using convolutional neural network for evaluation of maxillary sinusitis on panoramic radiography (Murata et al., 2019)	AI article	2019	48
Tooth detection and classification on panoramic radiographs for automatic dental chart filing: improved classification by multi-sized input data (Muramatsu et al., 2021)	AI article	2020^a^	28
Trabecular structure designation using fractal analysis technique on panoramic radiographs of patients with bisphosphonate intake: a preliminary study (Demiralp et al., 2019)	Non-AI article	2019	23
Evaluation of the mandibular trabecular bone in patients with bruxism using fractal analysis (Gulec et al., 2021)	Non-AI article	2021	22
A comparative study between CT, MRI, and intraoral US for the evaluation of the depth of invasion in early stage (T1/T2) tongue squamous cell carcinoma (Takamura et al., 2022)	Non-AI article	2021^a^	22
Artificial intelligence models for clinical usage in dentistry with a focus on dentomaxillofacial CBCT: a systematic review (Mureşanu et al., 2023)	AI review	2022^a^	21
Deep learning object detection of maxillary cyst-like lesions on panoramic radiographs: preliminary study (Watanabe et al., 2021)	AI article	2020^a^	19
Deep-learning approach for caries detection and segmentation on dental bitewing radiographs (Bayrakdar et al., 2022)	AI article	2021^a^	19
The relationship between COVID-19 and the dental damage stage determined by radiological examination (Sirin and Ozcelik, 2021)	Non-AI article	2021	18
Diagnostic charting of panoramic radiography using deep-learning artificial intelligence system (Başaran et al., 2022)	AI article	2021^a^	18
Evaluation of osseous changes in dental panoramic radiography of thalassemia patients using mandibular indexes and fractal size analysis (Bayrak et al., 2020)	Non-AI article	2020	18
Deep learning-based apical lesion segmentation from panoramic radiographs (Song et al., 2022)	AI article	2022	26
Evaluation of maxillary sinusitis from panoramic radiographs and cone-beam computed tomographic images using a convolutional neural network (Serindere et al., 2022)	AI article	2022	18
Comparison of multi-label U-net and mask R-CNN for panoramic radiograph segmentation to detect periodontitis (Widyaningrum et al., 2022)	AI article	2022	16
Automatic detection of periodontal compromised teeth in digital panoramic radiographs using faster regional convolutional neural networks (Thanathornwong and Suebnukarn, 2020)	AI article	2020	16
A deep learning approach to permanent tooth germ detection on pediatric panoramic radiographs (Kaya et al., 2022)	AI article	2022	15
Transfer learning in a deep convolutional neural network for implant fixture classification: a pilot study (Kim et al., 2022)	AI article	2022	12
A fully deep learning model for the automatic identification of cephalometric landmarks (Kim et al., 2021)	AI article	2021	12
Determining the reliability of diagnosis and treatment using artificial intelligence software with panoramic radiographs (Orhan et al., 2023)	AI article	2023	11
Correlation between gray values of cone-beam computed tomograms and Hounsfield units of computed tomograms: a systematic review and meta-analysis (Selvaraj et al., 2022)	Non-AI review	2022	10
Evaluation of the posterior superior alveolar artery canal by cone-beam computed tomography in a sample of the Egyptian population (Fayek et al., 2021)	Non-AI article	2021	10
Cone-beam computed tomography artifacts in the presence of dental implants and associated factors: an integrative review (Terrabuio et al., 2021)	Non-AI review	2021	10
Photoacoustic imaging of occlusal incipient caries in the visible and near-infrared range (Da Silva et al., 2021)	Non-AI article	2021	10
Oral surgery, oral medicine, oral pathology and oral radiology (limited to papers from the radiology section)			
Application of a fully deep convolutional neural network to the automation of tooth segmentation on panoramic radiographs (Lee et al., 2020)	AI article	2020	52
Contrast-enhanced computed tomography image assessment of cervical lymph node metastasis in patients with oral cancer by using a deep learning system of artificial intelligence (Ariji et al., 2019a)	AI article	2019	46
Automatic detection and classification of radiolucent lesions in the mandible on panoramic radiographs using a deep learning object detection technique (Ariji et al., 2019b)	AI article	2019	42
Deep learning systems for detecting and classifying the presence of impacted supernumerary teeth in the maxillary incisor region on panoramic radiographs (Kuwada et al., 2020)	AI article	2020	29
Applications of deep learning in dentistry (Corbella et al., 2021)	AI review	2021	26
An artificial intelligence system using machine-learning for automatic detection and classification of dental restorations in panoramic radiography (Abdalla-Aslan et al., 2020)	AI article	2020	25
Automated feature detection in dental periapical radiographs by using deep learning (Khan et al., 2021)	AI article	2021	24
Comparison of three deep learning neural networks for classifying the relationship between the mandibular third molar and the mandibular canal on panoramic radiographs (Fukuda et al., 2020a)	AI article	2020	18
Development of a radiomics and machine learning model for predicting occult cervical lymph node metastasis in patients with tongue cancer (Kubo et al., 2022)	AI article	2022	17
Automatic visualization of the mandibular canal in relation to an impacted mandibular third molar on panoramic radiographs using deep learning segmentation and transfer learning techniques (Ariji et al., 2022)	AI article	2022	17

^a^These papers were published online (early access) 1 year earlier than finally in print. The “publication year” listed here corresponds to the early access year recognized by JCR and official JIF calculation.

## 4 Discussion

The findings of this study show that AI papers were associated with higher JIF-accountable citation rates compared to non-AI papers. Since 2020, AI work, especially reviews synthesizing diagnostic tasks (e.g., lesion detection, landmarking) and modalities (panoramic, CBCT, CT), has yielded disproportionately high per-item citations in OMFR journals, suggesting high reader demand for state-of-the-art AI perspectives and practical synthesis for clinical imaging. AI papers have both methodological novelty and translational promise within OMFR. Reviews aggregate fast-moving evidence, while original AI articles often address high-impact tasks that are relevant to both researchers and clinicians, thus amplifying citations. The current findings echo with the notion that AI is one of the latest disruptive innovations in OMFR after digital imaging and CBCT/3D imaging (Joda et al., 2021; Tuygunov et al., 2025). The global momentum in AI development also likely contributes to increased dissemination and referencing of related work (Parteka and Kordalska, 2023).

There are both editorial and clinical implications of these findings for OMFR journals. The first issue is “signal vs. noise.” Since AI items can elevate journal-level metrics, editors should emphasize rigor and clinical relevance (e.g., curated datasets, external validation, reader studies) to prevent a novelty-driven publication skew. Second, reporting standards should be fostered, by encouraging adherence to imaging-AI reporting elements (e.g., transparent data partitions, appropriate reference standards, pre-specified endpoints) and requiring task-specific clinical readouts to improve real-world interpretability. Third, a balanced portfolio should be maintained by a healthy mix of AI and non-AI imaging content (e.g., CBCT protocols, MRI/US applications, quality assurance) so the journal portfolio remains comprehensive and avoids single-topic inflation. Four, structured state-of-the-science reviews and method primers (e.g., segmentation metrics, calibration pitfalls, domain shift) tightly anchored to OMFR clinical tasks can be commissioned.

From the current data, only a few AI papers were published in 2019 and 2020 and they received unusually high JIF-accountable citations, leading to very elevated notional JIFs for OMFR journals during those years. As the volume of AI publications increased substantially in subsequent years, citations became distributed across a larger set of papers, which naturally reduced the per-paper citation intensity and narrowed the gap between actual and notional JIFs. This pattern reflects the typical maturation of a rapidly expanding research domain and underscores the value of maintaining rigorous methodological and clinical standards as the field continues to grow. A follow-up analysis should be performed 5 or 10 years later to reveal if AI papers still generally receive more citations than non-AI counterparts.

This study has several limitations that warrant consideration. First, the manual classification of papers into AI and non-AI categories, despite high inter-observer agreement, may misclassify papers with borderline or implicit AI content. Second, it focused exclusively on OMFR journals, limiting the generalizability of the findings. Third, the analysis omitted journal editorial policies, open access status, and international collaboration status. Four, notional JIFs are illustrative and hypothetical. Finally, the statistical tests used could only demonstrate an association but not causality between JIF and the increasing publication and citation counts of AI papers. Since citation counts are typically skewed, the ANOVA results should be interpreted cautiously. Future work may apply mixed-effects or negative-binomial models to a large-scale dataset for more stringent inferential hypothesis testing, but this is beyond the scope of the current field-mapping study. Future research could expand the scope to other dental specialties and medical radiology, incorporate altmetric data, evaluate the impact of different types of AI models, and explore longitudinal trends in AI research.

## 5 Conclusion

AI-related publications exhibit higher citation density in OMFR journals, reflecting the characteristic of a rapidly evolving technological field. As AI research continues to expand, maintaining robust methodological standards and balanced editorial practices will support meaningful and sustainable scientific contributions.

## Data Availability

Publicly available datasets were analyzed in this study. This data can be found at: jcr.clarivate.com/jcr/home.
