# Supplementary material for: How artificial intelligence is reshaping citation impact in oral and maxillofacial radiology journals: an 8-year analysis with editorial and clinical implications (JCR 2017–2024)
Source: Front Res Metr Anal. 2026 May 8;11:1815503. doi: 10.3389/frma.2026.1815503 (PMC13194112; doi:10.3389/frma.2026.1815503)
Supplement: Supplementary file 1 [file Table_1.docx]

Supplementary Table S1. Raw numerator and denominator for each notional JIF.

| Journal | JCR Year | 2017 | 2018 | 2019 | 2020 | 2021 | 2022 | 2023 | 2024 |
| --- | --- | --- | --- | --- | --- | --- | --- | --- | --- |
| DMFR | Citation no. | 0 | 1 | 2 | 60 | 146 | 169 | 184 | 339 |
|  | Paper no. | 0 | 1 | 1 | 4 | 8 | 14 | 29 | 39 |
|  | Notional JIF | NA | 1.0 | 2.0 | 15.0 | 18.3 | 12.1 | 6.3 | 8.7 |
|  | 95% C.I. | NA | NA | NA | 7.6, 22.4 | 10.3, 26.3 | 5.2, 18.9 | 4.9, 7.8 | 6.5, 10.9 |
| Oral Radiol | Citation no. | 0 | 3 | 0 | 19 | 73 | 105 | 46 | 73 |
|  | Paper no. | 0 | 1 | 0 | 1 | 6 | 10 | 11 | 15 |
|  | Notional JIF | NA | 3.0 | NA | 19.0 | 12.2 | 10.5 | 4.2 | 4.9 |
|  | 95% C.I. | NA | NA | NA | NA | 3.0, 21.3 | 4.7, 16.3 | 1.8, 6.6 | 2.8, 6.9 |
| ISI | Citation no. |  |  |  |  |  | 42 | 52 | 91 |
|  | Paper no. |  |  |  |  |  | 8 | 11 | 13 |
|  | Notional JIF |  |  |  |  |  | 5.3 | 4.7 | 7.2 |
|  | 95% C.I. |  |  |  |  |  | 1.9, 8.6 | 3.3, 6.1 | 4.5, 9.8 |
| OOOO (radiology) | Citation no. | 0 | 0 | 0 | 40 | 86 | 127 | 62 | 48 |
|  | Paper no. | 0 | 0 | 0 | 2 | 6 | 9 | 9 | 6 |
|  | Notional JIF | NA | NA | NA | 20.0 | 14.3 | 14.1 | 6.9 | 8.0 |
|  | 95% C.I. | NA | NA | NA | NA | 7.8, 20.9 | 7.7, 20.6 | 4.0, 9.8 | 5.2, 10.8 |

Citation no., number of JIF-accountable citations received by citable items classified as AI papers (articles or reviews).

Paper no., number of citable items classified as AI papers (articles or reviews).
